# Electroconvulsive Treatment for Depression Alters Mitochondrial Serum Metabolites

**DOI:** 10.1016/j.bpsgos.2026.100754

**Published:** 2026-05-12

**Authors:** Erik Pålsson, Robert Sigström, Alireza Salehi, Mattias Hedenström, Axel Nordenskjöld, Mikael Landén

**Affiliations:** a Institute of Neuroscience and Physiology, Sahlgrenska Academy at University of Gothenburg, Gothenburg, Sweden; b Department of Digital Health, Research Institutes of Sweden, Stockholm, Sweden; c Department of Chemistry, Umeå University, Umeå, Sweden; d University Health Care Research Center, Faculty of Medicine and Health, Örebro Universitet, Örebro, Sweden; e Department of Medical Epidemiology and Biostatistics, Karolinska Institutet, Stockholm, Sweden

**Keywords:** Clinical study, Electroconvulsive treatment, Energy metabolism, Major depression, Metabolomics

## Abstract

**Background:**

Electroconvulsive therapy (ECT) is the most effective treatment for severe and treatment-resistant depression, but its biological mechanisms remain poorly understood. Given the pivotal role of mitochondria in cellular energy metabolism and their proposed involvement in the pathology of depression, we aimed to investigate whether ECT alters mitochondrial metabolism.

**Methods:**

We included 102 patients with major depressive disorder referred for ECT at 7 Swedish hospitals. Fasting serum samples were collected at 3 time points: immediately before the first ECT session (T0), 30 minutes after the first session (T1), and before the sixth session (T2). Proton nuclear magnetic resonance spectroscopy was used to quantify metabolites related to the tricarboxylic acid cycle and amino acid metabolism.

**Results:**

Acutely (T0→T1), serum levels of citrate, glucose, glutamine, and pyruvate increased significantly, while formate and phenylalanine decreased. Across the treatment course (T0→T2), alanine and pyruvate levels increased, whereas the ketone bodies acetoacetate, acetone, and 3-hydroxybutyrate decreased significantly. An exploratory analysis indicated that the reduction in ketone bodies (T0→T2) was confined to patients showing clinical improvement, as defined by the Clinical Global Impressions-Improvement scale.

**Conclusions:**

ECT induces both acute and sustained alterations in mitochondrial energy metabolism. These findings suggest that ECT modulates systemic mitochondrial function, warranting further investigation into how these metabolic changes relate to clinical improvement.

## Plain Language Summary

In this study, we examined whether ECT, an effective treatment for severe depression, affects the body’s energy metabolism. In 102 patients, blood samples showed both immediate and longer-term changes in metabolites linked to mitochondrial function after ECT. Notably, some energy-related metabolites increased shortly after treatment, while others changed over time. These findings suggest that ECT may influence how the body produces and uses energy, offering new clues to its biological effects, although further research is needed to understand how these changes relate to the clinical effect of ECT.

Major depressive disorder is a common condition associated with substantial disability (1) and societal costs (2). The clinical presentation of depression ranges from mild symptoms to severe, incapacitating episodes that require hospitalization. For mild to moderate depression, first-line treatment includes psychotherapy and/or antidepressant medication. For severe depression, particularly when accompanied by psychotic features, electroconvulsive therapy (ECT) is highly effective (3,4). However, the biological mechanism of action underlying the clinical effect of ECT remains poorly understood, and a significant proportion of patients do not achieve remission. Elucidating the biological effects of ECT could help identify biological markers predictive of treatment response and provide insights into its mechanism of action. Previous work has suggested that inflammatory (5) and microRNA–based biomarkers (6,7) may be useful in predicting clinical response to ECT.

Low energy, tiredness, and fatigue are cardinal symptoms of depression. Several studies implicate mitochondrial bioenergetics in the pathophysiology of psychiatric disorders, including major depressive disorder and bipolar disorder (8, 9, 10, 11, 12). Conversely, individuals with mitochondrial disorders experience psychiatric illnesses at a higher rate than the general population, and psychiatric symptoms often precede the diagnosis of mitochondrial disease (13). Mitochondria not only play a pivotal role in cellular energy metabolism but also participate in amino acid, lipid, and steroid metabolism (14), processes essential for synaptic signaling and brain plasticity. It is conceivable that one mechanism of action of ECT involves the regulation of mitochondrial metabolism, ultimately promoting neuronal functioning (15).

Serum metabolites associated with mitochondrial metabolism can be quantified using proton nuclear magnetic resonance (^1^H-NMR) spectroscopy in solution. ^1^H-NMR spectroscopy is a powerful technique for metabolic profiling of biofluids (16) and has previously been applied to identify potential biomarkers for central nervous system diseases (17) and neuropsychiatric disorders (18).

Our aim in the current study was to determine whether ECT influences mitochondrial function in patients with depression using a repeated-measures design. We used ^1^H-NMR metabolomics to quantify tricarboxylic acid (TCA) cycle metabolites and amino acids in serum samples collected 30 minutes before and 30 minutes after the first ECT session, as well as 30 minutes before the sixth ECT session. We further explored whether changes in TCA cycle metabolites were associated with clinical treatment response.

## Methods and Materials

### Study Population

Patients >18 years of age who were scheduled for a course of ECT for any indication and able to give informed consent were eligible for inclusion in the PREFECT (Predictors for Electroconvulsive Treatment) study. Patients were recruited between 2014 and 2016 from 7 psychiatric hospitals in Sweden: Danderyd, Huddinge, Hudiksvall, Sahlgrenska, Umeå, Uppsala, and Örebro. In Sweden, ECT is typically considered for inpatients with severe or treatment-refractory depression (defined as failure to respond to at least 2 classes of antidepressant medications), high suicide risk, or psychotic symptoms. The analyses and results presented here only include participants whose indication for ECT was a major depressive episode. All participants provided both oral and written informed consent. The PREFECT study was approved by the Regional Ethical Review Board in Stockholm (Reference No. 2012/1969-31/1).

### Study Design

Blood samples were collected at 3 time points: T0 (30 minutes before the first treatment session), T1 (30 minutes after the first session), and T2 (30 minutes before the sixth session). These time points were selected to capture both the acute metabolic effects of a single ECT session (T0→T1) and the longer-term changes occurring over the course of treatment unrelated to acute effects of an ECT session (T0→T2). The sixth session was chosen for the third sampling point (T2) because most patients with depression receive at least 6 ECT sessions.

### ECT Procedure

ECT was administered using bidirectional constant-current brief-pulse devices (Mecta; Mecta Corp. or Thymatron, Somatics Inc.). In Sweden, ECT is conducted according to national clinical guidelines (19) that recommend 3 ECT sessions per week using brief-pulse stimulation and right unilateral electrode placement. Succinylcholine (0.5–1.0 mg/kg) was used as a muscle relaxant, and glycopyrrolate (0.2 mg) or atropine was administered as an anticholinergic agent when needed. Through linkage to the Swedish National Quality Register for ECT (Q-ECT), we obtained data on the number of sessions in each treatment series, stimulation parameters for the first session, and the anesthetic agent used.

### Clinical Variables

Information on participant characteristics and medication use was obtained through linkage to the Q-ECT. The indication for ECT is recorded by the referring psychiatrist according to ICD-10 codes. For this study, we included patients whose indication was a major depressive episode in the context of major depressive disorder or bipolar disorder. Classification was based on ICD-10 codes and free text as described previously (20).

Within 1 week after ECT, clinicians also rated improvement using the Clinical Global Impressions-Improvement (CGI-I) scale (21), which ranges from 1 (very much improved) to 7 (very much worse). In this study, participants rated as very much improved or much improved were classified as responders, while participants with all other ratings were classified as nonresponders.

### Sample Collection and Serum Preparation

Fasting blood samples were drawn and collected in 10 mL serum tubes (Becton, Dickinson and Company). Samples were allowed to clot for 30 to 60 minutes at room temperature and then centrifuged (Heraeus Labofuge 200; Thermo Fisher Scientific) for 15 minutes at 2000*g*. Following centrifugation, serum aliquots were stored locally at the participating hospitals at −20 °C for a maximum of 30 days before transport to the Karolinska Institutet Biobank, where they were stored at −80 °C pending analysis.

### Sample Preparation and ^1^H-NMR Acquisition

For ^1^H-NMR analyses, serum samples were thawed and transferred from Eppendorf tubes to cryovials. Aliquots of 200 μL serum were mixed with 400 μL buffer (phosphate-buffered saline with 10% D_2_O, NaN_3_, and 5.5 mM trimethylsilyl propionate [TMSP]; pH 7.4) directly into 5 mm SampleJet NMR tubes (Bruker) using a Bruker SamplePro liquid handling system. Both the cryovials and the NMR tubes were kept at 4 °C during sample preparation, and mixing was performed directly in the SampleJet tubes using a 2-step mixing cycle.

The proton spectra were acquired at 37 °C on a Bruker 600 MHz Avance III HD spectrometer equipped with a 5-mm TCI cryoprobe and a SampleJet sample changer. T_2_ relaxation–edited spectra were recorded using a Carr-Purcell-Meibom-Gill pulse sequence with a total spin-lock time of 100 ms to attenuate broad signals from proteins and lipids. Water suppression was achieved using excitation sculpting. For each sample, 64 scans were collected with 64,000 data points, a spectral width of 14 ppm, and a relaxation delay of 1.1 seconds. The resulting free induction decays (65,536 data points each) were zero filled, and an exponential line–broadening function of 0.3 Hz was applied prior to Fourier transformation. All spectra were manually phase corrected and calibrated using the TMSP resonance at 0 ppm. Spectral processing was performed using Topspin 3.1 (Bruker BioSpin).

### Data Processing of ^1^H-NMR Spectra and Metabolite Identification

The spectra were prepared for statistical analysis by manually dividing them into 98 regions, each containing one or, in crowded regions, several peaks. These regions were individually aligned using an in-house MATLAB (version 2014b; The MathWorks, Inc.) script to minimize variability caused by minor peak shifts due to differences in pH, salt concentration, and temperature (22). The aligned regions were baseline corrected and then integrated, and the resulting peak areas were used for subsequent statistical analyses. Metabolite identification was accomplished using a combination of spectral line fitting in Chenomx 8.0 NMR software (http://www.chenomx.com) and reference spectra from the Human Metabolome Database (http://www.hmdb.ca).

### Statistics

We used a generalized least squares (GLS) model to estimate differences in metabolite levels at T1 and T2 relative to baseline (T0). The GLS approach accounts for data clustering caused by repeated measurements within individuals (23). We used an unstructured correlation matrix and a constant variance function, using the *corrSym* and *varIdent* functions in the R package *nlme* (version 3.1–167) (24). Before analysis, all NMR values of each metabolite were standardized to their mean value at T0 to ensure comparability across metabolites. *p* Values were adjusted for multiple testing using a 5% false discovery rate.

In exploratory post hoc analyses, we examined metabolomic changes in relation to sex and clinical response. First, we performed the analysis described above separately for responders and nonresponders and for men and women.

## Results

### Participant Characteristics

Blood samples from 107 participants were available for NMR acquisition. We excluded individuals without samples at any of the 3 time points (*n* = 1) and those with technical issues during NMR analysis (*n* = 4). After these exclusions, 102 participants remained in the final sample following NMR acquisition and data processing. Clinical and demographic characteristics of the study population are presented in Table 1.

**Table 1:** Sample Characteristics

	Overall, *n* = 102	Missing
Age, Years	45.0 (17.2)	0
Sex, Female	61 (59.8%)	0
Diagnosis		
Bipolar depression	27 (26.5%)	0
Major depressive disorder	75 (73.5%)	
MADRS-S Score		
Before ECT	34.7 (8.0)	20
After ECT	17.7 (12.2)	35
Remission, MADRS	23 (34.3%)	35
Clinical Improvement, CGI-I	73 (75.3%)	5
Number of ECT Sessions	8.8 (2.6)	0
Treatment Parameters at First Session		
Bilateral ECT	11 (10.9%)	1
Pulse width, ms	0.5 (0.1)	1
Electric charge, mC	228.5 (80.2)	1
Seizure time, EEG, s	47.3 (23.8)	1
Anesthesiologic Agent		
Propofol	25 (27.2%)	10
Tiopenthal	63 (68.5%)	
Other	4 (4.3%)	
Medication at First ECT Session		
Lithium	17 (16.8%)	1
Valproic acid	4 (4.0%)	1
Lamotrigine	9 (8.9%)	1
Second-generation antipsychotic	44 (43.6%)	1
First-generation antipsychotic	5 (5.0%)	1
Antidepressant	80 (79.2%)	1
No medication	6 (5.9%)	1

Values are presented as mean (SD) or *n* (%).

CGI-I, Clinical Global Impressions-Improvement; ECT, electroconvulsive therapy; EEG, electroencephalography; MADRS-S, Montgomery–Åsberg Depression Rating Scale, Self-Assessment.

### Changes in Serum Metabolites Following ECT

**Figure 1:**
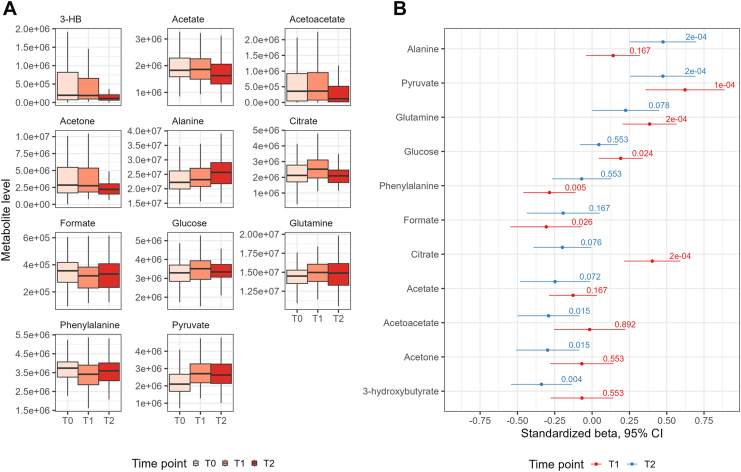
Metabolite level changes during electroconvulsive therapy (ECT). **(A)** Metabolite levels at T0 (immediately before first ECT), T1 (within 30 minutes after first ECT), and T2 (immediately before sixth ECT). **(B)** Estimated differences from T0 to T1 and T0 to T2, analyzed with generalized least squares models. Metabolites are sorted in order of their effect at T2. *p* Values are adjusted for a false discovery rate of 5%. 3-HB, 3-hydroxybutyrate.

**Figure 2:**
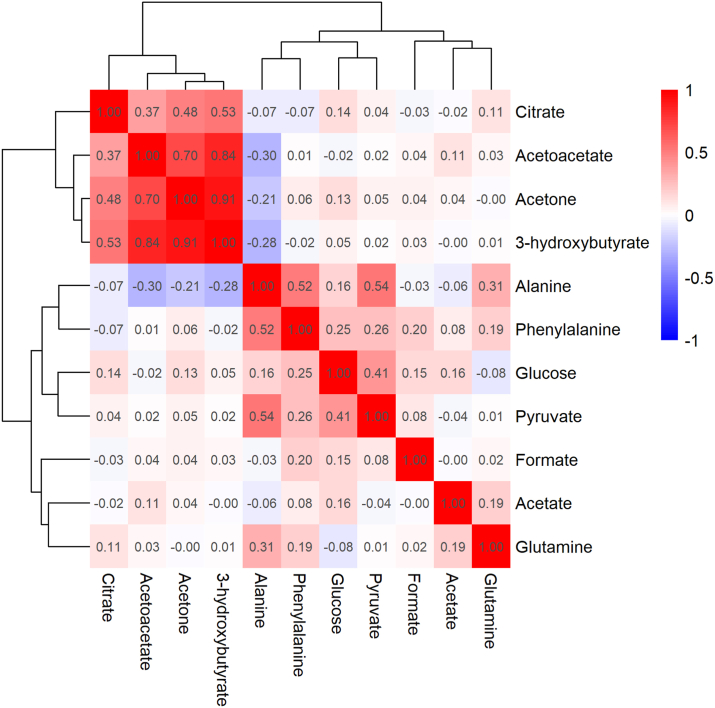
Correlation of metabolites before electroconvulsive therapy (ECT). Clustered heatmap displaying Pearson correlations between metabolites before ECT.

Figure 1 and Table S1 display serum metabolite peak areas at each sampling time point. Mean peak areas at T0 were compared with those at T1 and T2, respectively. Several metabolites showed statistically significant changes following ECT. Between T0 and T1, serum concentrations of citrate (*p* = .0002), glucose (*p* = .024), glutamine (*p* = .0002), and pyruvate (*p* = .0001) increased, while formate (*p* = .026) and phenylalanine (*p* = .0048) decreased. At T2, alanine (*p* = .0002) and pyruvate (*p* = .0002) concentrations were higher compared with T0, whereas acetoacetate (*p* = .015), acetone (*p* = .0002), and 3-hydrobutyrate (*p* = .0044) were lower. Figure 2 shows correlations between metabolites, age, and female sex at T0. The strongest correlations between metabolites were found for acetoacetate, acetone, and 3-hydrobutyrate.

### Serum Metabolites in Relation to Clinical Response to ECT and Sex

**Figure 3:**
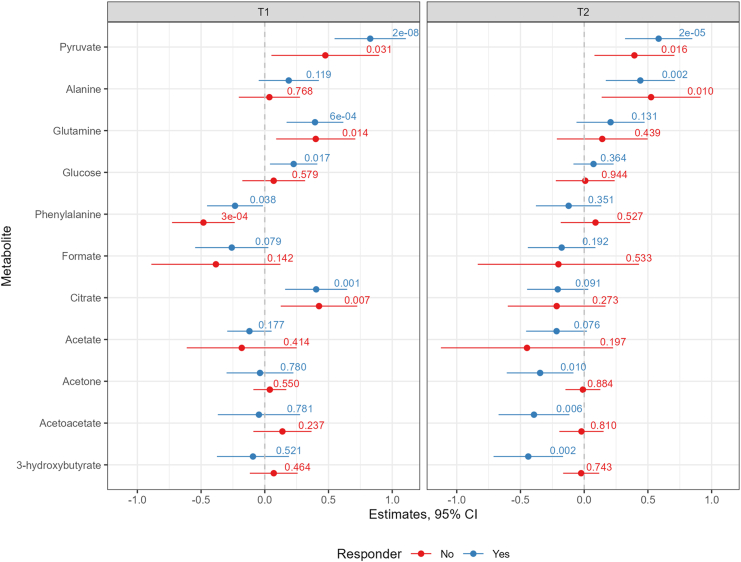
Exploratory analysis of changes in metabolite levels in responders and nonresponders to electroconvulsive therapy (ECT). Changes in metabolites in responders to ECT (Clinical Global Impressions-Improvement rating much improved or very much improved, *n* = 73) vs. nonresponders (all other ratings, *n* = 24). Estimates are standardized with 95% CIs. Numbers in the figure are uncorrected *p* values. Metabolites are ordered by their effect at T2 in responders. Results from generalized least squares models.

Among participants with available outcome data (*n* = 97), we examined changes in metabolites separately for responders to ECT (much improved or very much improved on the CGI-I, *n* = 73) and nonresponders (all other CGI-I ratings, *n* = 24). As this was an exploratory post hoc analysis, *p* values were not adjusted for multiple testing. As shown in Figure 3 and Table S2, there were no significant differences between responders and nonresponders at T1. At T2, however, only responders showed statistically significant decreases in 3-hydroxybutyrate (*p* = .002), acetoacetate (*p* = .006), and acetone (*p* = .01). We also examined changes in metabolites in men and women separately, showing generally similar changes in both sexes (Figure S1).

## Discussion

We analyzed blood samples from 102 patients with depression undergoing ECT using NMR spectroscopy to investigate whether ECT influences serum metabolites associated with mitochondrial energy metabolism. We found that concentrations of citrate, glucose, glutamine, and pyruvate increased immediately after the first ECT session (T0→T1), whereas formate and phenylalanine decreased. The pattern of changes differed over the course of treatment (T0→T2): Pyruvate concentration remained higher compared with baseline, while the other acute effects were not sustained. Instead, alanine concentration increased, and levels of acetoacetate, acetone, and 3-hydrobutyrate concentrations were lower at T2 compared with at baseline.

Serum citrate and pyruvate concentrations rose immediately after the first ECT session. These acute changes indicate seizure-induced increases in energy demand and substrate flux. However, acute changes in the flux of metabolic substrates may occur without increases in mitochondrial energy metabolism. Thus, acute changes in blood concentration of metabolic substrates are difficult to interpret in terms of mitochondrial energy metabolism. Pyruvate and citrate are early intermediates in the TCA cycle: Pyruvate can be converted to oxaloacetate by pyruvate carboxylase, which then condenses with acetyl-CoA to form citrate. Alternatively, pyruvate can be converted by pyruvate dehydrogenase to acetyl-CoA, which also enters the cycle by condensing with oxaloacetate to form citrate. Notably, pyruvate, but not citrate, remained significantly elevated above baseline at T2, suggesting that ECT may induce longer-lasting enhancement of mitochondrial energy conversion. Supporting this interpretation, electroconvulsive shock therapy in rats has been shown to increase the activity of mitochondrial respiratory chain enzymes (15).

A previous meta-analysis reported lower blood pyruvate levels in antidepressant-free patients with major depressive disorder (25). Although no difference was observed among patients receiving antidepressants, a more recent study also observed lower pyruvate concentrations in remitted patients compared with healthy control participants (26). Thus, while reduced pyruvate levels are associated with depression, their relationship with symptom remission remains unclear. Interestingly, a placebo-controlled trial of low-dose pyruvate supplementation in overweight individuals found improved mood in the active treatment group (27), suggesting that pyruvate alone may exert mood-enhancing effects. The mechanism by which ECT induces sustained increase in serum pyruvate is unknown, but possible explanations include modulation of the mitochondrial pyruvate–transporting system (28) or increased activity of mitochondrial enzymes involved in pyruvate metabolism (26).

Pyruvate is also a precursor to alanine, which is an alternative substrate in the TCA cycle. We observed that ECT resulted in a sustained—but not acute—increase in serum alanine levels. A previous study similarly reported an increase in plasma alanine peaking 24 hours after a single ECT session (29). Previous work has shown that both the L- and D-stereoisomers of alanine are present in human fluids and tissues (30). Although D-alanine is present at very low concentrations, it may still be relevant for psychiatric disorders (31). As our measurements do not permit differentiation between alanine isoforms, it is unclear whether the observed changes reflect alterations in L-alanine, D-alanine, or both. Alanine, together with glutamine, is an important glucogenic amino acid that contributes to glucose production (32). In the current study, both glutamine and glucose increased acutely after ECT, although these effects were not sustained over the course of treatment.

Changes in metabolite concentrations at T1 are likely to reflect the immediate physiological effects of ECT, whereas changes at T2 may result either from cumulative effects of ECT or from secondary processes related to symptom remission. Regardless of their origin, alterations at either time point could serve as potential biomarkers if they correlate with treatment response. Therefore, in an exploratory analysis, we tested whether changes in metabolite concentrations were associated with the clinical outcome of ECT. Because not all patients had data on treatment outcome, and the analysis was underpowered to detect interaction effects, we assessed associations by comparing responders and nonresponders separately.

We observed that decreases in acetoacetate, acetone, and 3-hydrobutyrate at T2 appeared to be linked to a favorable clinical response.

This could be interpreted as a metabolic shift away from lipid oxidation in ECT responders; however, the relationship between circulating ketones and depression is complex. A previous study using UK Biobank data reported a positive association between circulating ketone bodies and risk of depression (33). In contrast, other studies have suggested that a higher dietary ketogenic ratio is associated with fewer depressive symptoms (34) and that increased ketone levels from a ketogenic diet may have antidepressant effects (35). Thus, the observed association between lower acetoacetate, acetone, and 3-hydrobutyrate and clinical improvement following ECT does not necessarily imply a direct causal relationship. An increase in blood ketone levels is a metabolic hallmark of starvation (36). Loss of appetite and reduced energy intake leading to significant weight loss are typical features of melancholic depression. As melancholic features are strong predictors of ECT response (37), an increase in food intake during recovery could partially account for the changes in ketone body concentration observed at T2.

### Strengths and Limitations

This was a comparatively large study with repeated blood sampling, allowing examination of both acute and sustained effects of ECT on metabolism. We used ^1^H-NMR spectroscopy in solution, a sensitive analytical method for detecting proton-containing metabolites in biological samples. However, several limitations should be considered.

First, ECT involves both general anesthesia and neuromuscular blockade. Therefore, anesthetic agents might have contributed to the acute changes in serum metabolites. However, anesthesia is less likely to account for the sustained effects (T0→T2), as both samples were collected before anesthesia induction, although a cumulative carryover effect cannot be ruled out.

Second, information on medication use was available only at baseline. Although most patients continue the same psychotropic regimen during an ECT course, medication adjustments might have occurred and could have influenced the T0→T2 comparisons.

Third, although the analyzed metabolites can cross the blood-brain barrier, our measurements were based on serum samples, and we did not directly assess brain metabolism. Therefore, we cannot distinguish between metabolic changes occurring within the brain and systemic alterations secondary to clinical improvement or other mechanisms.

Fourth, the exploratory analyses of metabolite changes in relation to clinical response and sex were underpowered, and replication in larger samples is warranted.

Finally, only a subset of metabolites involved in energy metabolism were identified from the NMR data. Additional metabolites may also be affected by ECT, limiting the conclusions that can be drawn about its effects on mitochondrial metabolism. For example, glutamine is readily converted to glutamate in the glutamate-glutamine cycle. Previous work has highlighted the importance of measuring glutamate and glutamine separately when studying major depressive disorder treatment response (38). We measured only glutamine, which limits our ability to fully characterize glutamatergic metabolism.

### Conclusions

ECT was associated with both acute and sustained changes in serum metabolites related to mitochondrial energy metabolism. Exploratory analyses suggested that reductions in ketone bodies were restricted to patients who showed clinical improvement. Although these findings warrant replication, they support the notion that ECT affects systemic metabolic pathways linked to mitochondrial function. Further studies are needed to clarify whether these metabolic changes are related to the therapeutic effects of ECT and how they are related to other potential biomarkers of treatment response.

## Supplementary Material

Figure S1 and Tables S1–S2

Key Resources Table
