# Supplementary material for: Electroconvulsive Treatment for Depression Alters Mitochondrial Serum Metabolites
Source: Biol Psychiatry Glob Open Sci. 2026 May 12;6(5):100754. doi: 10.1016/j.bpsgos.2026.100754 (PMC13284473; doi:10.1016/j.bpsgos.2026.100754)
Supplement: Figure S1 and Tables S1–S2 [file mmc1.pdf]

SUPPLEMENTARY INFORMATION

Electroconvulsive Treatment for Depression Alters Mitochondrial Serum Metabolites

Pålsson *et al.*

Supplementary table 1. Metabolite levels and differences in metabolite levels at T1 and T2 vs. T0.

Metabolite	Time point	Mean	s.d.	Standardized difference	s.e.	Adjusted p-value
Acetate	T0	2069784	998024	Ref.		
	T1	1942319	645985	-0.130	0.082	0.17
	T2	1822330	813614	-0.250	0.120	0.072
Acetoacetate	T0	937248	1788137	Ref.		
	T1	907696	1694890	-0.017	0.120	0.89
	T2	416170	757366	-0.290	0.110	0.015
Acetone	T0	5044156	6988392	Ref.		
	T1	4564100	4999556	-0.069	0.110	0.55
	T2	2964817	2975542	-0.300	0.110	0.015
Alanine	T0	23184827	4950329	Ref.		
	T1	23882471	4712757	0.140	0.093	0.17
	T2	25537128	5024126	0.480	0.110	2e-04
Citrate	T0	2287685	855410	Ref.		
	T1	2632375	885504	0.400	0.096	2e-04
	T2	2117648	572691	-0.200	0.099	0.076
Formate	T0	357956	120579	Ref.		
	T1	320961	110903	-0.310	0.120	0.026
	T2	334626	119023	-0.190	0.120	0.17
Glucose	T0	3436667	1175413	Ref.		
	T1	3662108	1309811	0.190	0.075	0.024
	T2	3489963	1035493	0.045	0.065	0.55
Glutamine	T0	14183660	1983610	Ref.		
	T1	14948054	1799520	0.390	0.092	2e-04
	T2	14629961	1891453	0.220	0.110	0.078
Phenylalanine	T0	3666017	757533	Ref.		
	T1	3449697	776215	-0.290	0.089	0.0048
	T2	3613256	746557	-0.070	0.100	0.55
Pyruvate	T0	2306036	1044560	Ref.		
	T1	2956155	1225891	0.620	0.140	1e-04
	T2	2801336	1060990	0.470	0.110	2e-04
3-hydroxybutyrate	T0	676644	1309712	Ref.		
	T1	587976	969911	-0.068	0.110	0.55
	T2	232781	369894	-0.340	0.100	0.0044

Standardized differences indicate change in metabolite levels at T1 (within 30 minutes after first ECT) and T2 (immediately before sixth ECT) compared to T0 (immediately before first ECT). Analyses from generalized squares models. P-values are adjusted for a false discovery rate of 5%.
s.d.: standard deviation, s.e.: standard error

Supplementary table 2. Metabolite changes during ECT in responders and non-responders

		Responders (N=73)			Non-responders (N=24)		
Metabolite	Time point	Estimate	95% CI	p-value	Estimate	95% CI	p-value
Acetate	T1	-0.12	-0.29-0.05	0.177	-0.18	-0.61-0.25	0.414
Acetate	T2	-0.22	-0.46-0.02	0.076	-0.45	-1.12-0.23	0.197
Acetoacetate	T1	-0.05	-0.37-0.28	0.781	0.14	-0.09-0.37	0.237
Acetoacetate	T2	-0.39	-0.67--0.12	0.006	-0.02	-0.19-0.15	0.810
Acetone	T1	-0.04	-0.3-0.22	0.780	0.04	-0.09-0.17	0.550
Acetone	T2	-0.35	-0.61--0.08	0.010	-0.01	-0.15-0.13	0.884
Alanine	T1	0.19	-0.05-0.42	0.119	0.04	-0.2-0.28	0.768
Alanine	T2	0.44	0.17-0.71	0.002	0.52	0.14-0.91	0.010
Citrate	T1	0.4	0.16-0.65	0.001	0.43	0.13-0.73	0.007
Citrate	T2	-0.21	-0.45-0.03	0.091	-0.22	-0.6-0.17	0.273
Formate	T1	-0.26	-0.55-0.03	0.079	-0.38	-0.89-0.12	0.142
Formate	T2	-0.18	-0.44-0.09	0.192	-0.2	-0.84-0.43	0.533
Glucose	T1	0.23	0.04-0.41	0.017	0.07	-0.18-0.32	0.579
Glucose	T2	0.07	-0.08-0.23	0.364	0.01	-0.22-0.24	0.944
Glutamine	T1	0.39	0.17-0.62	6e-04	0.4	0.09-0.71	0.014
Glutamine	T2	0.21	-0.06-0.47	0.131	0.14	-0.21-0.5	0.439
Phenylalanine	T1	-0.23	-0.45--0.01	0.038	-0.48	-0.73--0.24	3e-04
Phenylalanine	T2	-0.12	-0.38-0.13	0.351	0.09	-0.18-0.36	0.527
Pyruvate	T1	0.83	0.55-1.11	2e-08	0.47	0.05-0.9	0.031
Pyruvate	T2	0.58	0.32-0.85	2e-05	0.39	0.08-0.71	0.016
3-hydroxybutyrate	T1	-0.09	-0.38-0.19	0.521	0.07	-0.12-0.26	0.464
3-hydroxybutyrate	T2	-0.44	-0.71--0.17	0.002	-0.02	-0.17-0.12	0.743

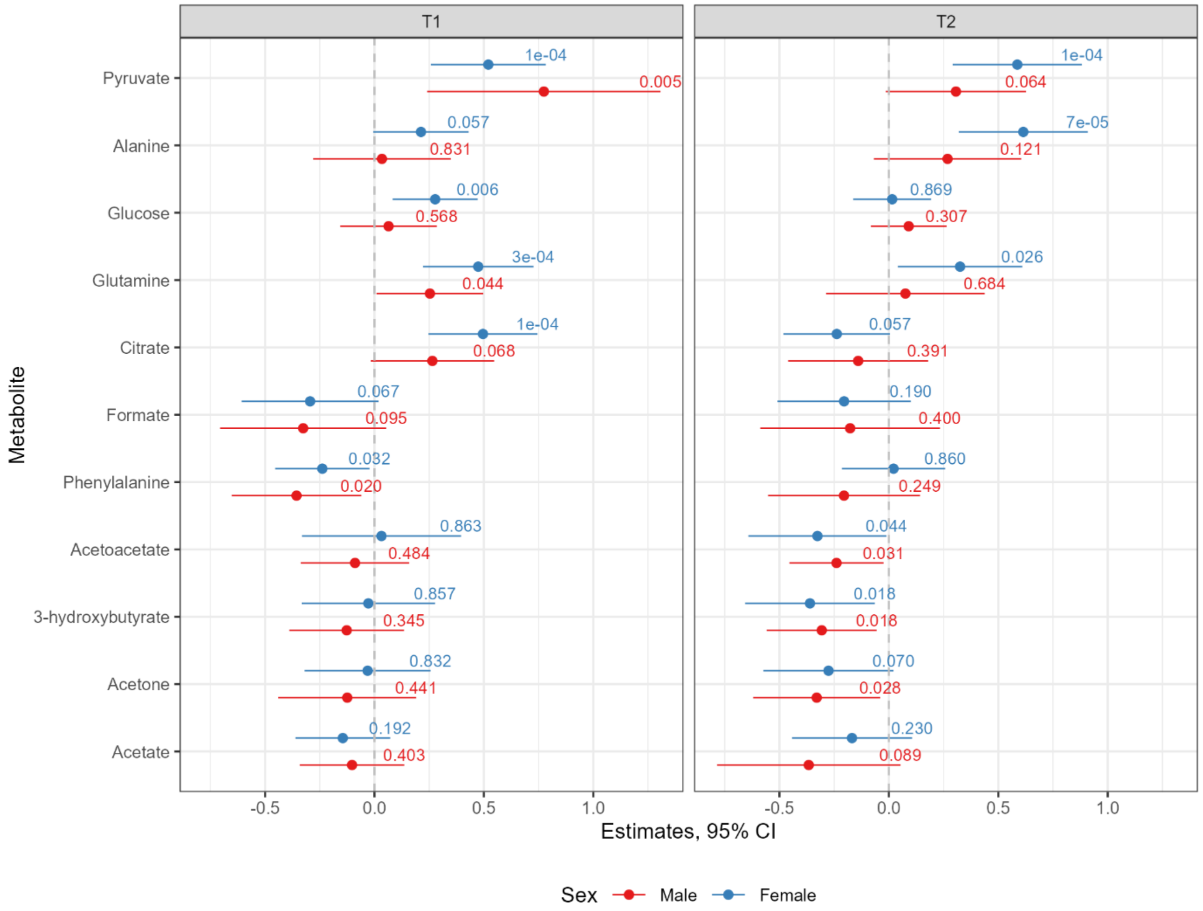
T1
T2
Pyruvate
Alanine
Glucose
Glutamine
Citrate
Formate
Phenylalanine
Acetoacetate
3-hydroxybutyrate
Acetone
Acetate
Metabolite
1e-04
0.005
0.057
0.831
0.006
0.568
3e-04
0.044
1e-04
0.068
0.067
0.095
0.032
0.020
0.863
0.484
0.857
0.345
0.832
0.441
0.192
0.403
1e-04
0.064
7e-05
0.121
0.869
0.307
0.026
0.684
0.057
0.391
0.190
0.400
0.860
0.249
0.044
0.031
0.018
0.018
0.070
0.028
0.230
0.089
-0.5
0.0
0.5
1.0
Estimates, 95% CI
Sex
Male
Female

Figure S1.
